# *Aloe vera*/Chitosan-Based Edible Film with Enhanced Antioxidant, Antimicrobial, Thermal, and Barrier Properties for Sustainable Food Preservation

**DOI:** 10.3390/polym16020242

**Published:** 2024-01-15

**Authors:** Navjot Kaur, Chandran Somasundram, Zuliana Razali, Abdel-Hamid I. Mourad, Fathalla Hamed, Zienab F. R. Ahmed

**Affiliations:** 1 Department of Integrative Agriculture, College of Agriculture and Veterinary Medicine, United Arab Emirates University, Al Ain 15551, United Arab Emirates; 202190077@uaeu.ac.ae; 2 Institute of Biological Sciences, Faculty of Science, University of Malaya, Kuala Lumpur 50603, Malaysia; chandran@um.edu.my (C.S.); zuliana@um.edu.my (Z.R.); 3 The Center for Research in Biotechnology for Agriculture (CEBAR), University of Malaya, Kuala Lumpur 50603, Malaysia; 4 Department of Mechanical and Aerospace Engineering, College of Engineering, United Arab Emirates University, Al Ain 15551, United Arab Emirates; ahmourad@uaeu.ac.ae; 5 Department of Physics, College of Science, United Arab Emirates University, Al Ain 15551, United Arab Emirates; fhamed@uaeu.ac.ae

**Keywords:** bioactive coating, *Ficus carica*, biopolymer, fresh produce, food packaging, plant-based film

## Abstract

Food bioactive packaging has received increasing attention from consumers and the food industry for its potential to reduce food waste and environmental issues. Several materials can be used to produce edible films/coats; however, bio-based, cost-effective, and sustainable coatings have gained a high reputation these days. For instance, *Aloe vera* gel (AV) is a promising bio-based material for edible coatings and films; therefore, the present study aimed to investigate the film-forming abilities of AV and Chitosan (CH) combination as a potential active food packaging material. The physicochemical and mechanical characteristics of formed films of various combinations were prepared at different concentrations, i.e., CH (0.5% *w*/*v*), AV (100%), CH:AV (75:25), and CH:AV (60:40). The results showed significant differences among all the prepared edible films wherein these differences were mainly on account of incorporating AV gel. The rheological and antioxidant properties of the formulations improved with the inclusion of AV gel. The films composed of CH:AV (60:40) positively affected the water solubility, thermal properties, and water vapour permeability of the edible films. The X-ray Diffraction (XRD) and Scanning electron microscopy (SEM) results showed that the films composed of CH:AV, (60:40) were amorphous and had smooth morphology. Further, the edible film solutions were applied to fresh figs (*Ficus carica*) to investigate their role in preserving fruits during storage. A significant reduction in microbial growth was found in coated fruits after 28 days of cold storage. The films composed of CH and AV showed overall improved results compared to the CH (0.5%, *w*/*v*). Therefore, the used formulations (CH:AV, 60:40) can form a sustainable film that has the potential to be utilized for fresh product preservation to maintain its quality and shelf life.

## 1. Introduction

The current approach is moving more towards sustainable solutions, which can be observed in consumers’ demand and acceptability for eco-friendly products. Plant-based sources such as edible coatings could be the best solution. Plant-based sources have been used as an extract or essential oil, requiring lengthy and costly downstream processing after getting the crude extract. Therefore, extensive research is going on to find excellent source material, that can be used alone or in combination with other edible coatings, and it emerged as an advantage for fresh produce industries as they have upraised the market value of their products by increasing their product validity. Since this approach protects fresh produce from external and internal injuries, provides safety, increases shelf life, and maintains quality, it is extensively utilized in food industries [1,2].

The term edible films and coatings are generally used interchangeably by many researchers. However, there is a technical distinction between both terms. Edible coatings are hydrocolloids that can be applied to the product using different techniques such as dipping, brushing, and spraying, upon drying they form a film around the product, whereas edible films are first developed into films using the liquid coating material and then adhere to the product surface [2,3,4]. The primary mechanism of action of edible films and coatings is to develop a thin layer or semi-permeable barrier around the product to minimize its contact with the external environment. This will subsequently create a protective and modified atmosphere around the fresh produce, which can preserve its freshness for a long time [3]. If the film develops a very thick or extremely thin layer on a product surface, it may produce undesired results. An important criterion used to develop optimum coating and films considers several parameters such as economic reliability, mechanical properties, thermal stability, sensory properties, and barrier properties. All these parameters are greatly influenced by the molecular weight and concentration of the material, the type of solvent used to prepare the coating, pH, temperature, and additives used. Therefore, this is challenging to formulate a coating or develop a film with the desired characteristics.

Several materials have been characterized for their film-forming abilities, including lipids (waxes or oils), proteins (gelatin or whey protein), biopolymers (alginate or chitosan), and carbohydrates (starches or cellulose derivatives) [4,5,6]. Each material has a unique composition and function [7]. Among these materials, Chitosan (β-(1–4)-2-acetamido-D-glucose and β-(1–4)-2-amino-D-glucose units) is the most widely practiced biopolymer in films and coatings-making processes. Its inert and biodegradable nature and antimicrobial and antioxidant properties make it superior among other coating materials [8,9].

CH is a natural cationic polysaccharide obtained from Chitin which naturally occurs in the hard shell of fungi, insects, lobster, shrimp, snails, and other sea animals. Chitin is converted to CH by demineralization, deproteinization, discoloration, and deacetylation. The molecular structure of CH is composed of hydroxyl groups, oxygen molecules, and amino groups, which give the CH ion exchange, flocculation, and viscous properties. Additionally, highly extended semi-crystalline structures in the CH molecule make dissolving in an aqueous solution more difficult. Therefore, it is best dissolved in weakly acidic solutions such as lactic acid or Acetic acid [10,11]. The structural properties of CH vary according to the degree of deacetylation, molecular weight, and product grade. Due to these variations, the applications of CH have become broader and extended to many different fields such as it is reported as an excellent base material for food packaging, cosmetics, wound dressing, tissue engineering, drug delivery, and adsorbent in wastewater remediation [8,12,13,14,15,16].

Despite its numerous advantages, CH still needs some improvements regarding its thermal and mechanical stability and water vapor permeability properties which are important factors of edible films and coatings [17]. Several studies reported that the films composed of pure CH are highly permeable to water vapor which is not a suitable requirement for food packaging industries, especially in humid environments [18,19]. To overcome these disadvantages, researchers have explored multiple ways of modifying the CH, out of which the blending of CH with other bio-based materials is considered promising. Combining such materials with CH has enhanced its thermal and mechanical stability and improved the antioxidant and antimicrobial properties of CH-based edible films and coatings [20,21]. It was reported that the properties of pure CH have been improved by combining it with other polymers and plant extracts such as starch, guar gum, alginate, carboxy methyl cellulose, gelatin, pineapple peel extract, lemon peel extract, basil essential oil [22,23,24,25,26,27,28].

Recently, various studies have reported the *Aloe vera* gel (*Aloe barbadensis* Miller) (AV) as an excellent base material for edible films and coatings [29,30,31]. It is well known for its therapeutic properties and has been widely used in cosmetics and herbal medicines [32]. It is a short perennial succulent xerophyte plant that belongs to the Aloaceae family with more than 500 species existing worldwide. It comprises about 12 to 16 leaves with a thick layer of epidermis surrounding the mesophyll cells. Underneath the mesophyll cells, transparent jelly like material is present, which is mainly composed of 98% of water, and the rest of the portion consists of carbohydrates, fibers, amino acids, proteins, soluble sugars, vitamins, minerals, organic acids, and several bioactive compounds [33,34]. The compatibility of AV gel has been found suitable with various biopolymers that make it an appropriate material for edible films [35,36,37,38,39].

The exploration of optimal film properties and their subsequent behavioral analysis on application-based platforms is imperative for discerning their inherent characteristics and potential impact. Engaging in such examinations is crucial to derive comprehensive insights into their performance, enabling a more nuanced understanding of their dynamic interactions and efficacies. Therefore, the objective of this study was to investigate the best formulation of AV and CH formulation that can develop optimal films to be used as coating materials for promoting the quality, shelf-life, and retard senescence and decay of various fresh horticulture produce during storage. The mechanical, thermal, rheological, and chemical bonds, surface morphology, and color properties of the formulation and the developed films will be investigated. The same solutions were evaluated to assess their efficacy in inhibiting microbial proliferation and prolonging the shelf life of fresh figs (*Ficus carica*). Though figs are highly nutritious and delicious, their extremely perishable nature and short shelf life limit their marketability. Considering their short shelf life, using them to evaluate the films would be a viable approach. Hence, the fruits were used to examine the effect of coatings in extending their postharvest lifespan. The films/coatings that will be developed in the current study will have the potential as active food packaging material, that can be employed to improve the shelf life and maintain the quality of fresh produce in a sustainable way.

## 2. Materials and Methods

### 2.1. Material

For this experiment, low molecular weight CH (chitin deacetylated ≥ 75%) and Methanol from Sigma–Aldrich Co (St. Louis, MO, USA), Peptone water, Plate count agar (PCA), and Potato dextrose agar (PDA) were bought from HiMedia Laboratories, 507 School House Rd., Kennett Square, PA, USA, 1,1-Diphenyl-2-picrylhydrazyl (DPPH), and 2,2′-azino-bis (3-ethylbenzothiazoline-6-sulfonic acid) (ABTS) were bought from TCI chemicals, Toshima, Tokyo, Japan and fresh AV leaves were harvested from the farm in the College of Agriculture and Veterinary Medicine, at United Arab Emirates University, Al Ain, UAE.

### 2.2. Aloe vera Gel Preparation

Fresh AV leaves with uniform size, color, and maturity were washed with distilled water to remove the dust from the outer epidermis and then kept at room temperature in a vertical position to release the yellow compound called Aloin. Afterwards, the peel was removed using a knife, and the transparent gel was collected using a sterile spatula. The gel was homogenized for 10 min to make a fine paste using IKA, T25 digital ULTRA TURRAX homogenizer (Str. 10, Staufen, Germany). Later, the homogenized gel was centrifuged for 10 min at 4 °C to remove the debris and the supernatant was then filtered using a muslin cloth to remove any remaining insoluble particles. The fine transparent AV gel was then pasteurized at 70 °C for 45 min in an oven [30].

### 2.3. Preparation of Edible Film Solutions

The solutions were prepared as mentioned in Table 1. The stock solution of 1% CH was prepared in 1% lactic acid in distilled water (*v*/*v*) and then used to make different formulations. The first edible film of CH alone was prepared at 0.5% (*w*/*v*) in 1% lactic acid in distilled water (*v*/*v*). Pure AV gel was considered as a second edible film solution and labelled as AV (100%). The third edible film solution was prepared by homogenizing 75 mL of CH (1%, *w*/*v*) with 25 mL of pure AV gel and labelled as CH:AV (75:25). The fourth edible film solution was prepared by homogenizing 60 mL of CH (1%, *w*/*v*) with 40 mL of pure AV gel and labelled as CH:AV (60:40). Homogenization time was set to 2–3 min for all the preparations and the homogeneity of the formulations was investigated by measuring the absorbance of solutions at 600 nm just after the preparation of solutions. All the formulations were kept stable for 24 h in sterile tubes. Afterward, absorbance from the lower and upper portions of the solution was taken to check the differences. The homogenous formulation was taken for further analysis.

#### 2.3.1. The Viscosity, pH, and Particle Size Measurement of Edible Films Solutions

The viscosity of the edible film solutions was measured in Centipoise (cP) using an Ametek viscometer, Brookfield, WI, USA. The freshly prepared solutions just before plating were used to measure the viscosity with concentric cylinder starch cell geometry using a gap of 100 µm at 25 °C and the set speed of 100 RPM [40]. The pH of the edible film solutions was measured using a pH meter. For measuring the particle size, zeta potential, and polydispersity index of the edible coating solutions, Dynamic Light Scattering (DLS) analyzer (Nanotrac Wave II, PA, USA). The refractive index of the samples was set to 1.7 in the dialog box of Microtrac Flex Analysis software version 12.0.1.1 [41]. All the above measurements were taken in three technical and biological replicates.

#### 2.3.2. Antioxidant Activity of Edible Film Solutions

A modified method was used to measure the antioxidant activity of all the solutions using 2,2-Diphenyl-1-picrylhydrazyl (DPPH), and 2,2′-azino-bis (3-ethylbenzothiazoline-6-sulfonic acid) (ABTS) free radicals [40,41]. For the DPPH assay, the DPPH stock solution (50 mg/100 mL) was prepared in absolute methanol. From the stock solution, a working solution of DPPH was prepared by dissolving 500 µL aliquot into 4.5 µL of absolute methanol. An aliquot of 500 µL from the sample was added to the DPPH working solution and kept in the dark for 45 min at room temperature. After 45 min, the absorbance of each sample was measured at 517 nm using a UV-visible spectrophotometer. Working solution without a sample was considered as a control. For the ABTS assay, the stock solutions of ABTS (7 mM) and potassium persulfate (2.6 mM) were prepared. The working solution of ABTS was prepared by mixing the above two stock solutions in a 1:1 ratio and left in the dark for 12 h to react with each other. Just prior to performing the assay, dilute the working solution of ABTS with 80% methanol to get the absorbance ~0.710 at 732 nm. The ABTS radical scavenging assay was performed by mixing 3 mL of diluted ABTS solution with 30 µL of sample. The mixture was allowed to react in the dark at room temperature for 6 min then the absorbance was measured immediately at 732 nm using a UV-visible spectrophotometer. ABTS diluted solution without sample was considered as control. The % DPPH and % ABTS scavenging was calculated using the following Equation (1):

(1) $$Scavenging\;(\%)=\frac{Absorbance\;of\;control-Absorbance\;of\;sample}{Absorbance\;of\;control}\times 100.$$

### 2.4. Preparation of Edible Films

Edible films were prepared using the casting method with some modifications [42]. A volume of 20 mL of each homogenous solution was poured into 90 mm Petri dishes and dried at room temperature. After drying, the films were peeled off using forceps and kept at ambient conditions (25 ± 2 °C and 55 ± 1% RH). The films were used immediately for the analysis.

#### 2.4.1. Color, Opacity, Water Solubility (WS), and Water Vapor Permeability (WVP) of Edible Films

The color parameters: L* (Lightness), a* (red, green), and b* (blue, yellow) of edible films were recorded using a Hunter Lab colorimeter (HunterLab–LabScan XE, Hunter Associates Laboratory Inc., Reston, VA, USA). The opacity of the films was measured by dividing the film’s thickness by the film’s absorbance (600 nm). The water solubility of edible films was measured according to the modified protocol [43]. Films were cut into small strips of size 2 × 2 cm and weighed to get initial weight in milligrams (mg), then they were subjected to drying in the oven for 24 h at 100 °C. After drying, the final weight (mg) was taken, and the films were left stirred into 15 mL of distilled water for 24 h at ambient temperature. Later the dissolved films in water were filtered using Whatman filter paper, and the filtrate was again dried to get the final dry weight of undissolved films. The weight of dried film strips used initially was considered initial dry weight. The water solubility (WS) percentage of films was calculated using the following Equation (2):

(2) $$WS\;\%=\frac{\left\{Initial\;dried\;weight\left(mg\right)-Final\;dried\;weight\left(mg\right)\right\}}{Initial\;dried\;weight\;(mg)}\times 100$$

The water vapor permeability of the films was calculated [44]. Small crucibles with an internal diameter of 30 mm and an outer diameter of 35 mm with a 30 mm depth were filled with 8 mL of distilled water. Using a plastic rubber band, each cup was sealed tightly with an edible film. The crucibles were weighed to record the initial weight. Then they were placed in a desiccator filled with silica gel to maintain a humidity level of 0%. After every 12 h for 3 consecutive days, the crucibles were weighed to record the weight change. The following equation was used to calculate the Water Vapor Permeability (WVP) (3):

(3) $$WVP\left({gm}^{-2}\;h^{-1}\;{Pa}^{-1}\;mm\right)=\frac{\Delta m\times X}{A\times t\times \Delta P},$$

where Δm is the change in mass (g), X is the film thickness (mm), A is the area of the film (m^2^), t is the time (h), and ΔP is the partial pressure difference of the atmosphere of water and the silica gel.

#### 2.4.2. Scanning Electron Microscopy (SEM) and X-ray Diffraction (XRD) of Edible Films

Film samples were examined for surface morphology using SEM (JSM-6010PLUS/LA, Jeol, Tokyo, Japan). The films were prepared twice from freshly prepared solutions. Small sections from three different areas of films were cut and used for capturing image. A total 9 pictures were taken for each film with different magnifications to cover all the area. The SEM images with 500× magnification were chosen. Gold coatings were applied to the film strips. The SEM investigations were carried out at an accelerating voltage of 5 kV. The crystalline nature of the films was examined by SHIMADZU Lab X–XRD–6100 diffractometer, Shimadzu Corp., Kyoto, Japan. The XRD was performed at Cu-Kα radiation, 40 kV accelerating voltage, and 15 mA current. XRD profiles were recorded at a scan rate of 2/min and scan range from 5 to 65.

#### 2.4.3. Mechanical Properties of Edible Films

Film thickness was measured in millimetres using a digital micrometer, and then film samples were cut into a specific specimen size with a gauge length of 3 cm. The Zwick–Roell tester with 5 kN load-cell (according to ASTM D882 standard [45]) was used with a test speed of 5 mm/min to get the force (N) and stroke (mm) values. These values were used to calculate the tensile strength (MPa), Strain %, and Young’s modulus (Mpa) of the films.

#### 2.4.4. Thermal Properties of Edible Films

A differential scanning calorimeter (DSC) was used to investigate the glass transition (Tg) and melting temperature (Tm) of the films. A temperature range of 0–350 °C at a 10 °C min^−1^ heating rate was employed to obtain the thermographs.

#### 2.4.5. Fourier Transform Infrared (FTIR) Spectroscopy of Edible Films

The samples’ functional groups were detected using the Thermo Fisher Scientific model Nicolet 6700 (Waltham, MA, USA). 16 scans at 1 cm^−1^ resolution varying from 1000 to 3551 cm^−1^ were run.

### 2.5. Application of Edible Film Solutions on Fresh Fig Fruits

Fig fruits (cv. Brown turkey) were collected from trees grown in open fields at the research center’s farm in Al Ain City, Abu Dhabi, UAE. Fruits were harvested at the commercial maturity stage and immediately transported to the laboratory. Fig fruits without defects, pests, or mechanical damage, and uniform (in color and size) were randomly selected and distributed into 4 groups (10 fruits per group) each for one treatment (Control (water), CH (0.5%, *w*/*v*), CH:AV (75:25), and CH:AV (60:40)). Fruits were given dip treatment for 60 s and then dried at room temperature. Afterward, fruits were placed in cold storage at 2 °C (90–95% R.H.) for up to 28 days.

The total fungal/mold and bacterial count on fruits coated with edible film solutions was determined before cold storage and at the end of the storage period of 28 days. One gram of fresh macerated fruit tissue was vortexed vigorously in 9 mL autoclaved buffered peptone water and many serial dilutions were prepared. One ml sample from each dilution was pour plated with plate count agar (PCA) media for the bacterial count and potato dextrose agar (PDA) for fungal/mold count. The whole experiment was performed under sterile conditions using a laminar Air flow chamber (LAF). To detect the bacterial growth, the plates were incubated at 37 °C for 48 h, and for fungal count the plates were incubated at 27 °C for 5 days. The number of colonies was recorded at the end of incubation time and calculated as Log10 colony forming unit per grams of fresh weight (Log_10_ CFU g^−1^). This experiment was performed two times in triplicate [12].

### 2.6. Statistical Analysis

All the measurements were taken in three biological and technical replicates, and the significant differences were checked using a one-way analysis of variance (ANOVA). Tukey’s test was applied to distinguish the formulations at *p* ≤ 0.05. The statistical analyses were performed using Minitab Statistical Software version 21 for the window program.

## 3. Results

The present study investigates the best formulation using AV and CH at different ratios as film coating materials. Various combinations were prepared to check the film-forming abilities. CH and AV solutions were prepared stand-alone as a baseline and in different combinations. After several rounds of trials, it was observed that the increasing concentration of AV tends to increase the brittleness of films. Hence, the present study has limited the AV concentration to 40%. Moreover, the AV gel (100%) alone did not form the film; therefore, the successful combinations that formed films were analyzed for the mechanical, thermal, chemical bonds, surface morphology, and color properties.

### 3.1. The Viscosity, pH, Zeta (ζ)-Potential, Polydispersity Index (PDI), and Particle Size

In the current study, significant differences (*p* ≤ 0.05) in the viscosity of CH (0.5%, *w*/*v*), AV (100%), CH:AV (75:25), and CH:AV (60:40) were detected with 161.5, 24.5, 53.2, and 43.4 cP, respectively (Table 2). Whereas, no significant difference was observed in the pH of films containing CH; however, the AV (100%) was found to be comparatively basic, and CH (0.5%, *w*/*v*) was slightly acidic. These results correlate with the charge density of edible film solutions, where CH (0.5%, *w*/*v*) showed the highest positive zeta potential (102.4 mV) followed by CH:AV (75:25) and CH:AV (60:40) with 68.2 mV and 56.6 mV, respectively (Table 2). In contrast, AV (100%) showed the lowest negative zeta potential (−42.95 mV). The particle size of CH in different formulations ranges from 2.29 µm to 1.43 µm. The CH:AV (75:25) has the highest particle size (2.29 µm) followed by CH (0.5%, *w*/*v*), CH:AV (60:40), AV (100%) with 2.13 µm, 1.43 µm, and 1.15 µm, respectively (Table 2). Similarly, CH (0.5%, *w*/*v*) and CH:AV (60:40) showed the lowest PDI as compared to the AV (100%) and CH:AV (75:25).

### 3.2. Antioxidant Property of Edible Film Solution

**Figure 1:**
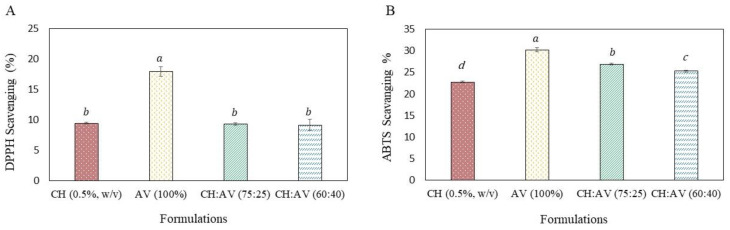
Antioxidant activity of edible films. (**A**) DPPH Scavenging (%) and (**B**) ABTS scavenging (%) of edible films solution. Bars with different superscript letters are significantly different (*p* ≤ 0.05). CH: Chitosan, AV: *Aloe vera* gel.

The antioxidant property of the film solutions was measured by DPPH radical scavenging assay and ABTS radical scavenging assay, and results are presented as DPPH scavenging (%) and ABTS scavenging (%), respectively (Figure 1A,B). In this case, the AV (100%) showed the highest DPPH scavenging percentage (17.95%); however, no significant difference was observed in the scavenging activity of CH (0.5%, *w*/*v*) (9.44%), CH:AV (75:25) (9.31%), and CH:AV (60:40) (9.16%) (Figure 1). A similar trend was observed in the ABTS radical scavenging assay where AV (100%) showed the highest antioxidant activity (30.2%), and CH alone showed the lowest antioxidant activity (22.7%).

### 3.3. Color, Opacity, Water Solubility (WS), and Water Vapor Permeability (WVP) Properties of Edible Films

In the current study, the color values (L*, a*, b*, and Chroma) of edible films were measured, and the CH (0.5%, *w*/*v*) films had the lightest color which was indicated by the highest L* value and lowest Chromatic value, as compared to the rest of the films. These results are correlated with the opacity value of the films which suggests that the addition of AV increased the opacity of the films and the films composed of CH (0.5%, *w*/*v*) were found least opaque (Table 3). The results of the film’s water solubility obtained in the present study are presented in Table 3. Films composed of CH and AV were least dissolved in the water (~40% solubility) as compared to the CH (0.5%, *w*/*v*) (60.3% solubility). The WVP of the films was affected by the addition of AV. It is observed that the incorporation of AV into the CH has significantly reduced the WVP of the edible films (Table 3). The CH (0.5%, *w*/*v*) showed the highest WVP with 0.267 gm^−2^ h^−1^ Pa^−1^ mm, followed by CH:AV (75:25) and CH:AV (40:60) with 0.202 and 0.082 gm^−2^ h^−1^ Pa^−1^ mm, respectively.

### 3.4. Scanning Electron Microscopy (SEM) and X-ray Diffraction (XRD) of Edible Films

**Figure 2:**
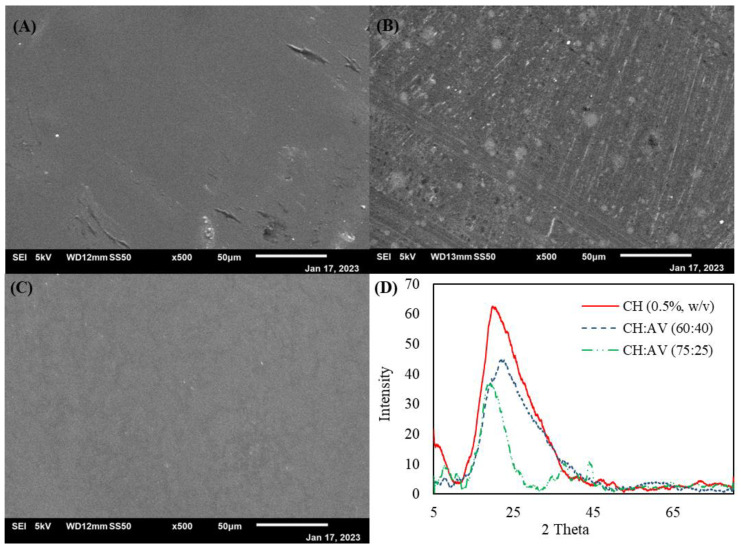
Scanning electron microscope images: (**A**) CH (0.5%, *w*/*v*), (**B**) CH:AV (75:25), and (**C**) CH:AV (60:40); and (**D**) XRD profiles of the edible films. CH: Chitosan, AV: *Aloe vera* gel.

The XRD profiles of the edible films composed of CH and AV gel are presented in (Figure 2D). The profiles indicate the amorphous nature of the edible films with a broad diffraction peak. The boarding of the diffraction peak decreased with the addition of AV and was more pronounced for CH:AV (75:25). This is a signal toward the beginning of the formation of small nanocrystals. Scanning electron microscopy results indicate that the films did not show any cracks or holes. The CH (0.5%, *w*/*v*) and CH:AV (60:40) showed smooth films, whereas the CH:AV (75:25) showed some overlapping pattern and roughness (Figure 2A–C).

### 3.5. Mechanical Properties

**Figure 3:**
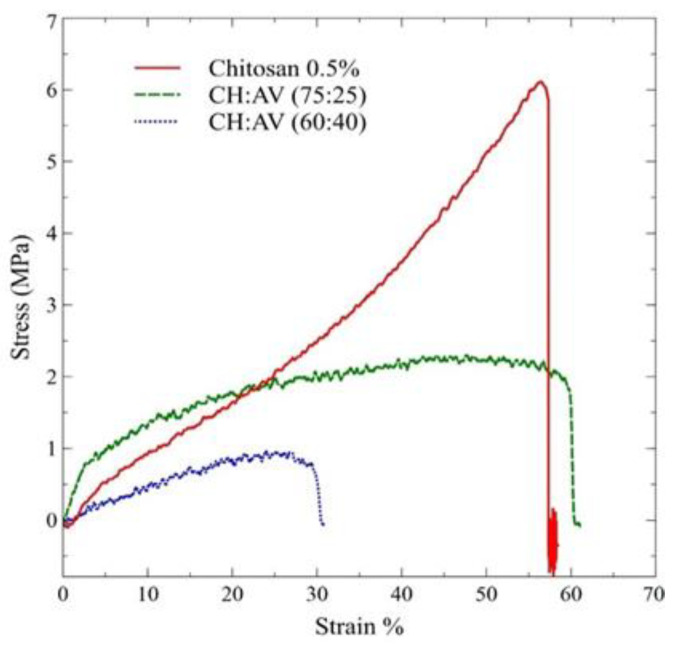
Stress v/s Strain curve of edible films. CH: Chitosan, AV: *Aloe vera* gel.

In the present study, the thickness of the films varies proportionally with the concentration of CH (Table 4). CH (0.5%, *w*/*v*) films showed the least thickness with 0.033 mm, followed by CH:AV (60:40) (0.042 mm), and CH:AV (75:25) (0.050 mm) in increasing order. The concentration of the film-forming solution, CH alone, was prepared with 0.5% (*w*/*v*), whereas the concentration of CH used for mixing with AV was prepared with 1% (*w*/*v*). Therefore, CH alone had the lowest CH concentration and CH:AV (75:25) had the highest CH concentration despite AV (Table 1). The film made of CH alone showed the highest tensile strength with 0.061 MPa, as compared to the CH:AV (75:25) (0.016 Mpa) and CH:AV (60:40) (0.001 Mpa) where the CH:AV (60:40) has the highest AV concentration as compared to CH:AV (75:25). A similar trend can be seen in Young’s modulus values in which CH (0.5%, *w*/*v*) (9.35 Mpa) has the highest value followed by CH:AV (75:25) (5.60 Mpa) and CH:AV (60:40) (3.61 Mpa) in decreasing order. Stress–strain curve for different film combinations were plotted as shown in (Figure 3). The results of this work showed a high Strain % in CH (0.5% *w*/*v*) (58.5%) and CH:AV (75:25) (63%) as compared to CH:AV (60:40) (33%).

### 3.6. Thermal Properties

**Figure 4:**
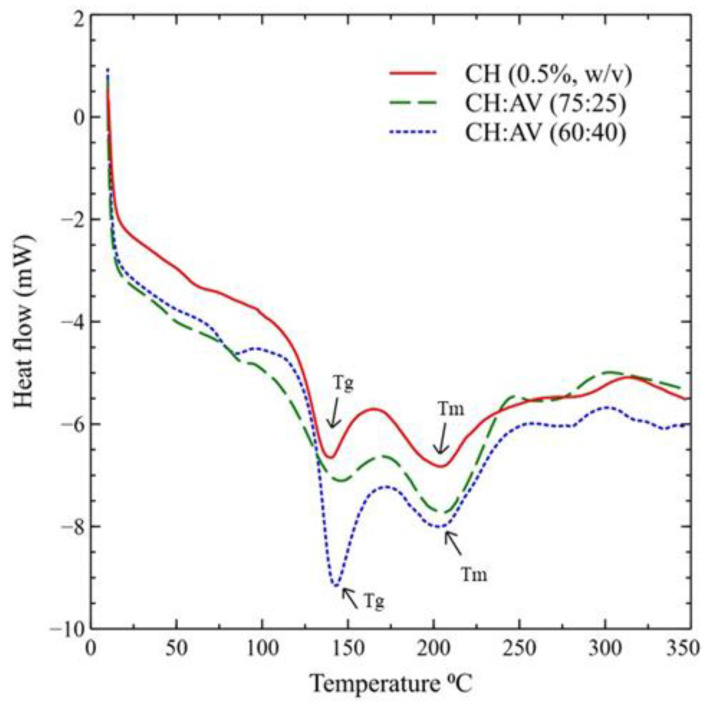
Differential Scanning Calorimetry graph of edible films. CH: Chitosan, AV: *Aloe vera* gel.

The thermal properties of the films were analyzed by DSC and the results are presented in (Figure 4). In the DSC graph, a minor peak at 62.53 °C, 88.48 °C, and 82.58 °C signifies the water molecules’ evaporation in the film samples. The next peak corresponds to the glass transition temperature of the CH (0.5%, *w*/*v*), CH:AV (75:25), and CH:AV (60:40) films, which were found to be 138.30 °C, 145.08 °C, and 142.43 °C, respectively. The melting temperature was detected in a range of 203 to 206 °C for all the films.

### 3.7. Fourier Transform Infrared (FTIR) Spectroscopy

**Figure 5:**
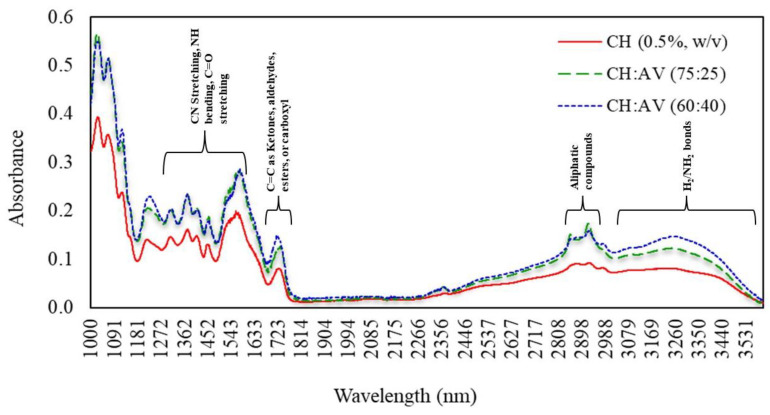
Fourier transforms infrared spectroscopy graph of edible films. CH: Chitosan, AV: *Aloe vera* gel.

The interaction between CH and AV gel has been analyzed and is presented in (Figure 5). The broad peak at 3000 cm^−1^ represents the bond stretching of O-H and N-H groups [46,47]. Two sharp peaks were observed in CH:AV (60:40) and CH:AV (75:24) films at 2855.8 and 2922.5 cm^−1^, indicating the presence of aliphatic compounds (CH_2_) [48]. Three characteristic peaks of CH can be observed at 1311 cm^−1^, 1560 cm^−1^, and 1650 cm^−1^, representing CN stretching, NH bending, and CO stretching, respectively. Another prominent peak at 1720 cm^−1^ could be due to carbonyl group vibration. The peak at 1379 cm^−1^ indicates the presence of the acetamide group of CH [49,50]. Several peaks of different functional groups attributed to the AV gel were observed at 1070, 1260, 1420, 1615, 1717, 2930, and 3274 cm^−1^ indicating the stretching and vibration of the carboxylic group (C-O), alkane group (CH_3_), aromatic group, CH=CHR group, aldehyde group (C=O), alkene group (CH_2_), and OH group, respectively [21,50,51].

### 3.8. Microbial Load on Fig Fruits

**Figure 6:**
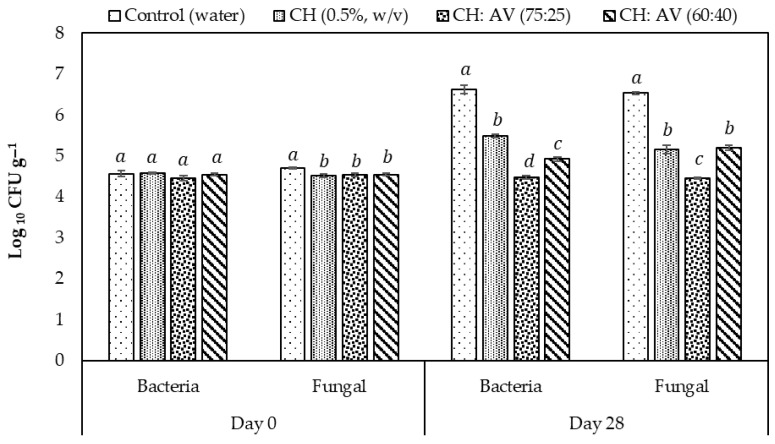
Effect of different edible coatings on microbial load on fruits during cold storage, values are the mean (n = 10) ± SE. Means with different letters on the bars for each treatment are significantly different at *p* > 0.05 using Tukey’s test.

The total bacterial count (TBC) and fungal count (TFC) on coated and uncoated fig fruits were measured before and after cold storage for 28 days and the results are presented in (Figure 6). Overall results declared no significant differences in the TBC and TFC at day 0; however, increased microbial growth was found in all the treatments except CH:AV (75:25) by the end of storage. The fruits coated with CH and AV had decreased bacterial and fungal growth as compared to the control. In the present study, it is observed that the CH:AV (75:25) treatment followed by CH:AV (60:40) and CH (0.5%, *w*/*v*) significantly retarded the microbial growth on fig fruits as compared to the control. Therefore, it can be concluded that the mentioned combination of CH and AV can be used to prevent microbial spoilage in fruits thereby extending the storage life.

## 4. Discussion

The viscosity of film-forming solutions is an important parameter that impacts their effectiveness in protecting food products and enhancing their shelf life. It is influenced by several factors, including the type and concentration of the materials used, the molecular weight and structure of the polymers, the temperature and pH of the solution, and the presence of other additives such as plasticizers or surfactants [10]. Highly viscous coating solutions may provide better adhesion and uniform film despite these being more difficult to apply and may result in a thick film that can alter the texture and taste of the food product. However, the lower viscous films are easier to apply but may not provide as much protection and can result in a thinner and less uniform coating [52,53]. The present study attempted to formulate a film-forming solution with optimum viscosity to make it feasible for effective film. The results indicate that adding AV gel has decreased the viscosity of the film solutions [54]. It has been reported that the viscosity of fresh AV gel decreases with the storage time due to the presence of a glucomannan compound that starts forming cross-linking bonds [55]. Since there are no significant differences found in the pH; therefore, the differences observed in viscosity cannot be interfered with by the pH factor. However, the structure of polymers itself might have played a role in the viscous properties of the formulations [10]. The higher the charge density of the solution, the more viscous the solution will be [56].

The intermolecular interactions within the solution can be studied by the magnitude of the zeta potential which provides information about the strength of the electrostatic repulsion or attraction between particles [57]. The higher the zeta potential value, the more the electrostatic repulsion within the particles in the solution. In comparison, less value indicates the non-stability of the particles in the solution, which can lead to aggregation and is more prone to peeling or cracking of the films. Ultimately affects the barrier properties of the films [56]. AV gel’s lower viscosity and zeta potential might be the reason for its incapability to form the film. Another important parameter is the particle size which affects a material’s physical, chemical, and biological properties. The particle size in a liquid solution can influence the performance of edible film used in the food industry. The film-forming properties, including adhesion to the entity’s surface, are highly modulated by the size of particles present in the used solutions [10]. Smaller particles have a larger surface area, which can make uniform and dense films to protect the food surface, whereas larger particles may lead to rough and non-uniform films. Moreover, small-size particles are promising in making smooth films that can provide good sensory properties to the food [58]. The particle size of CH in the solution can be affected by various factors such as concentration, pH, temperature, and ionic strength. Generally, in acidic conditions such as the 1% lactic acid solution, CH can form small aggregates or particles due to the protonation of its amino groups [56]. This could be the reason for higher particle size in the case of CH:AV (75:25). On the other hand, adding AV gel to the CH solution has reduced its particle size. Similarly, the stability and uniformity of the formulation solution were estimated by the polydispersity index, which measures the degree of heterogeneity or distribution of particle sizes in a sample. Generally, the low PDI indicates a narrow particle size distribution which ensures consistent film quality and thickness, which can lead to improved product shelf life and sensory characteristics. Moreover, narrow size distribution can also improve the stability of the coating emulsion or solution, reducing the risk of phase separation or instability during storage.

Films with antioxidant activity are a bonus that not only supports and improves its own life but the product also. Here we used AV gel which contains potential bioactive compounds with antioxidant activity, including phenylpropanoids and coumarins, flavonoids, phenylpyrone and phenol derivatives, and phytosterols [34]. The antioxidant activity can be detected by the reduction of the free radical form of DPPH and ABTS by antioxidants which can lead to a change in the color of DPPH from purple to yellow and of ABTS from dark green to light green. This color change can be measured as a decrease in absorbance. The higher the antioxidant amount in the sample, the lighter the color. AV gel was found with the highest antioxidant activity however, the blending of AV with CH has significantly affected the antioxidant properties of AV. This might be due to the hindrance of molecules in CH that reduced the availability of bioactive compounds present in AV. Therefore, an optimum concentration of CH is required to maintain the antioxidant property of AV.

Furthermore, the visual properties of edible films are the most important factor in consumers’ acceptability as they directly impact their choices. This can be qualified by measuring color parameters. In the present study, films composed of CH alone showed the lightest and almost transparent color whereas, the increasing concentration of AV has darkened the film color (light yellow). This might be due to the presence of anthraquinones (Aloin) which might have undergone a chemical transformation into a phenolic compound called Aloe amodin upon drying process [59]. A similar trend can be observed in the opacity of films where the films containing AV are more translucent than the rest. These results agree with those presented by [21]. Another crucial parameter of edible films is their water solubility, whose requirement varies depending on the application purpose; for example, less soluble films are preferred during food storage, whereas soluble films are preferred for instant food [60]. Here we found films incorporated with AV gel were less soluble as compared to the CH alone. Reduced solubility of the films with increasing concentrations of AV was found in one report [44]. They also found 100% solubility of the films made of pure CH. The results of water solubility are highly correlated with the results obtained in the WVP. WVP defines the release of moisture through the hydrophilic part of the film matrix [19]. Due to the hydrophilic nature of CH, it has more tendency to let the water molecules transverse through its membrane. However, the results indicate that its incorporation with AV has reduced its tendency. This might be due to a decrease in the availability of hydrophilic groups in CH due to the overlapping interactions of the compounds present in the AV [44,61]. Hence, it can be concluded that the addition of AV to the CH has significantly improved the barrier properties of edible films. Therefore, films composed of AV and CH can be applied to the food items to extend the storage life as they are less soluble and less permeable as compared to the CH alone.

On the other hand, the structural properties of the films were analyzed using scanning microscope and XRD. The XRD profiles of the films showed similar results with only one prominent broad peak, which could not declare the crystal nature of the films as the rest of the graph has no significant peaks. This indicates the amorphous nature of the films. A peak at 2θ (19–20°) is attributed to the CH and coincides with the “Form II” crystal pattern [11,62]. The influence of AV gel incorporated into CH can be observed as a slight shift in the peak of the 2θ angle. Adding AV gel to CH (1%) in a 40:60 ratio did not affect the film’s surface morphology. A study found that the increasing concentration of AV by up to 30% has increased the roughness of the films [23,63]. This can be correlated to the results obtained in the present study in the case of films composed of CH and AV in a ratio of 75:25 which showed slight roughness in surface morphology compared to the rest of the films. However, the CH and AV in the ratio of 60:40 improved the film quality. Hence, the results of XRD and SEM presented in the current study indicate that the films made of CH (0.5%, *w*/*v*) and CH and AV gel in a ratio of 60:40 are amorphous in nature and, therefore, can be considered as a good material for film formation. The crystal lattice structure determines the peak sharpness at a particular angle. The broadening of the peaks decreased in the case of CH films incorporated with AV gel, signifying a decline in the amorphous structural state [21,63]. Similar results were found roughness in the film surface upon adding 15% of AV gel to the films [21].

The functionality of edible films is highly dependent on their mechanical properties, such as tensile strength, elongation at break, and modulus of elasticity [64,65,66,67]. For packaging and coating applications, the mechanical properties of the films play a significant role. The optimal range of film thickness relies on the application purposes; for example, thinner films are preferred in the packaging industries due to the less material usage and better oxygen or moisture barrier properties, while thicker films provide many advantages for coating applications by maintaining the structural integrity of fresh produce [3,68]. Several factors influence the thickness of the film, such as temperature, duration, and concentration of the material used [69]. The results signify that the thickness of the edible films increased with the increasing concentration of CH, whereas AV did not play many roles in increasing the film thickness. Certain factors modulate the strength of the films, including the presence of additives or processing aids, the degree of cross-linking or crystallinity, and the temperature and humidity conditions during testing [69]. The results obtained in the current study indicate that the incorporation of AV gel has reduced the tensile strength of the films. Similar observations were reported wherein the addition of AV decreased the mechanical properties of the film [54,70,71]. Contrasting results were obtained in another study which reported an increase in the tensile strength of the films with an increase in the AV gel concentration [72]. This occurred due to the incorporation of alginate, which cross-linked with AV and strengthened the film. Another important parameter of mechanical properties is Strain %, which also allows the material to resist deformation and failure under tension. The Strain % depends on the flexibility and durability of the material. For example, materials with higher crystallinity have lower Strain % [73]. The results of this work showed a high Strain % in CH (0.5%, *w*/*v*) (0.60%) and CH:AV (75:25) (0.63%) as compared to CH:AV (60:40) (0.33%). This might be due to the higher concentration of AV in CH:AV (60:40) which might have increased the brittleness of the film nature. The rest of the formulations have higher CH concentrations which indicates that the CH films are flexible and less fragile [17]. Generally, the material with high tensile strength and Young’s modulus, but low Strain % possesses higher cross-linkage, regardless of the thickness of the material. The results suggest that the concentration of AV and CH has a significant impact on the mechanical properties of the edible films.

DSC was performed to investigate the thermal properties (Glass transition temperature (Tg) and melting temperature (Tm)) of the edible films. These properties are crucial to analyze the film changes due to the temperature effect. The most important factors are the glass transition temperature and melting temperature, which describe the thermal and mechanical stability of the films. The glass transition temperature tells the transformation of films from a glassy state to a relaxed state, while the melting temperature is the temperature at which films begin to melt [74,75]. The results showed that the addition of AV has slightly increased the glass transition temperature of the films. This might be due to the weakening of intermolecular forces between CH molecules [76]. The results obtained in the present study showed higher glass transition temperatures than those reported by [21]. Moreover, the higher concentration of CH used in the CH:AV (75:25) and CH:AV (60:40) has also led to a higher glass transition temperature [77,78]. The Tm of the films detected in the current study is greater than the results mentioned by Wang et al. (2023) [20]. This might be due to the different methods used for the preparation of film using different plasticizers. An endothermic peak in the range of 203 to 206 °C describes the melting temperature of the films which is far away from the optimal temperature conditions for food packaging as well as for storing the horticulture produce. Based on the current results, the edible films composed of CH and AV gel can sustain a high temperature of about 200 °C.

Functional groups present in the films were identified using Fourier transform infrared (FTIR) spectroscopy. Different functional groups possess different spectral behaviour, which can further address the variations in the polymers at the molecular level [48]. Using this advanced technology, several peaks were identified at different wavelengths ranging from 1300 to 1800 cm^−1^ and 2800 to 3000 cm^−1^, which signifies the presence of various bonds. Furthermore, these peaks suggest that the incorporation of AV gel did not interfere with the molecular structure of CH. Therefore, it can be concluded from the present results that the addition of AV gel to the CH or visa-versa has not changed the chemical properties of any of them, and hence, the differences between these three films are majorly related to absorption peaks regardless of the wavelength.

The application of edible film solutions on fresh fig fruits was completed to evaluate their performance in prolonging the fruit’s shelf life. Figs are very delicate and can easily undergo mechanical damage due to postharvest handling and storage conditions. These damages help microorganisms to adhere and spoil the fruit [79]. Microbiological assessment of fruits during cold storage for 28 days clearly differentiates the coated fruits from uncoated fruits. CH has been reported to possess antimicrobial properties and has a role in the jasmonic acid pathway. Previous studies mentioned the role of CH in providing resistance against *Botrytis cinerea* in ripened strawberries and grapes. This is a common fungus causing postharvest degradation in fruits [79]. Likewise, AV gel has antioxidant and antimicrobial properties that resist the growth of microorganisms [30]. The results suggested that the incorporation of AV had enhanced the antimicrobial effect of the coating material. Therefore, AV and CH-based coatings around the fruit can protect it from microbial degradation and enhance its shelf life.

## 5. Conclusions

The results obtained in the current study suggested that the performance of edible films composed of Chitosan has improved by combining with *Aloe vera* gel. A significant improvement was observed in the melting temperature, surface morphology, water solubility, and water vapor permeability of the film CH:AV (60:40). These improvements can be seen in the Zeta potential, Polydispersity index, Particle size, thermal stability, SEM, XRD, FTIR, water solubility, and water permeable properties. Further investigations related to the application of these film-forming solutions and their effect on the shelf life and quality of fresh produce declared their role in preventing microbial spoilage thereby extending the shelf life. The overall results suggest that the film developed in the current study has the potential as an active food coating/packaging material that can be employed to improve the shelf life and maintain quality.

## Figures and Tables

**Table 1:** Formulations of edible film formed from Chitosan and *Aloe vera*-based solutions.

Formulations	Percentage of CH in Film Solution	Percentage of AV in Film Solution
CH (0.5%, *w*/*v*)	0.5%, *w*/*v*	Nil
AV (100%)	Nil	100% (Pure)
CH:AV (75:25)	0.75%, *w*/*v*	25%, *v*/*v*
CH:AV (60:40)	0.60%, *w*/*v*	40%, *v*/*v*

CH: Chitosan, AV: *Aloe vera* gel.

**Table 2:** The ζ-potential, polydispersity index (PDI), particle size, viscosity, and pH of the edible film solutions.

Formulations	Zeta Potential (mV)	Polydispersity Index (%)	Particle Size (µm)	Viscosity (cP)	pH
CH (0.5%, *w*/*v*)	102.4 ± 0.70 ^a^	0.01 ± 0.00 ^b^	2.13 ± 0.00 ^a^	161.5 ± 1.49 ^a^	3.37 ± 0.05 ^b^
AV (100%)	−42.95 ± 0.55 ^d^	0.95 ± 0.35 ^ab^	1.15 ± 0.12 ^b^	24.5 ± 0.50 ^d^	4.39 ± 0.03 ^a^
CH:AV (75:25)	68.2 ± 1.26 ^b^	1.25 ± 0.16 ^a^	2.29 ± 0.06 ^a^	53.2 ± 1.38 ^b^	3.43 ± 0.08 ^b^
CH:AV (60:40)	56.6 ± 0.90 ^c^	0.08 ± 0.00 ^b^	1.43 ± 0.03 ^b^	43.4 ± 0.53 ^c^	3.41 ± 0.70 ^b^

Values are presented as mean ± SE, n = 3. Values with different superscript letters are significantly different (*p* ≤ 0.05). CH: Chitosan, AV: *Aloe vera* gel.

**Table 3:** Color, Opacity, Water solubility, and Water vapor permeability properties of edible films.

Formulations	L*	a*	b*	Chroma	Opacity (%)	WS (%)	WVP (gm^−2^ h^−1^ Pa^−1^ mm)
CH (0.5%, *w*/*v*)	12.75 ± 1.53 ^c^	−0.24 ± 0.01 ^a^	0.34 ± 0.97 ^b^	0.25 ± 0.96 ^c^	1.17 ± 0.64 ^c^	60.32 ± 0.52 ^a^	0.267 ± 0.07 ^a^
CH:AV (75:25)	17.68 ± 1.26 ^b^	−0.07 + 0.05 ^c^	1.35 ± 0.06 ^a^	0.41 ± 0.83 ^b^	1.84 ± 0.57 ^b^	40.74 ± 0.65 ^b^	0.202 ± 0.17 ^b^
CH:AV (60:40)	23.31 ± 0.73 ^a^	−0.18 ± 0.01 ^b^	0.19 ± 0.12 ^c^	1.35 ± 0.76 ^a^	2.36 ± 0.86 ^a^	39.10 ± 0.64 ^b^	0.082 ± 0.11 ^c^

Values are presented as mean ± SE. Values with different superscript letters are significantly different (*p* ≤ 0.05). CH: Chitosan, AV: *Aloe vera* gel, WS: Water solubility, WVP: water vapor permeability.

**Table 4:** Mechanical properties of edible films.

Formulations	Tensile Strength (Mpa)	Strain (%)	Young Modulus (Mpa)	Film Thickness (mm)
CH (0.5%, *w*/*v*)	0.061 ± 0.41 ^a^	58.5 ± 0.01 ^a^	9.35 ± 0.59 ^a^	0.033 ± 0.001 ^c^
CH:AV (75:25)	0.016 ± 0.50 ^b^	63.0 ± 0.07 ^a^	5.60 ± 1.69 ^ab^	0.050 ± 0.002 ^a^
CH:AV (60:40)	0.006 ± 0.21 ^b^	33.0 ± 0.02 ^b^	3.61 ± 0.25 ^b^	0.042 ± 0.001 ^b^

Values are presented as mean ± SE. Values with different superscripts are significantly different (*p* ≤ 0.05). CH: Chitosan, AV: *Aloe vera* gel.

## Data Availability

Data are contained within the article.
